# Long-term culture and significant expansion of human Sertoli cells whilst maintaining stable global phenotype and AKT and SMAD1/5 activation

**DOI:** 10.1186/s12964-015-0101-2

**Published:** 2015-03-25

**Authors:** Ying Guo, Yanan Hai, Chencheng Yao, Zheng Chen, Jingmei Hou, Zheng Li, Zuping He

**Affiliations:** State Key Laboratory of Oncogenes and Related Genes, Renji-Med X Clinical Stem Cell Research Center, Ren Ji Hospital, School of Medicine, Shanghai Jiao Tong University, 160 Pujian Road, Shanghai, 200127 China; Department of Urology, Ren Ji Hospital, School of Medicine, Shanghai Jiao Tong University, Shanghai Institute of Andrology, Shanghai Human Sperm Bank, 145 Shangdong Road, Shanghai, 200001 China; Shanghai Key Laboratory of Assisted Reproduction and Reproductive Genetics, Shanghai, 200127 China; Shanghai Key Laboratory of Reproductive Medicine, Shanghai, 200025 China

**Keywords:** Human Sertoli cells, Isolation & identification, Long-term culture, Significant expansion, Global gene profiles, AKT and SMAD1/5 signaling pathways

## Abstract

**Background:**

Sertoli cells play key roles in regulating spermatogenesis and testis development by providing structural and nutritional supports. Recent studies demonstrate that Sertoli cells can be converted into functional neural stem cells. Adult Sertoli cells have previously been considered the terminally differentiated cells with a fixed and unmodifiable population after puberty. However, this concept has been challenged. Since the number of adult human Sertoli cells is limited, it is essential to culture these cells for a long period and expand them to obtain sufficient cells for their basic research and clinic applications. Nevertheless, the studies on human Sertoli cells are restricted, because it is difficult to get access to human testis tissues.

**Results:**

Here we isolated adult human Sertoli cells with a high purity and viability from obstructive azoospermia patients with normal spermatogenesis. Adult human Sertoli cells were cultured with DMEM/F12 and fetal bovine serum for 2 months, and they could be expanded with a 59,049-fold increase of cell numbers. Morphology, phenotypic characteristics, and the signaling pathways of adult human Sertoli cells from different passages were compared. Significantly, adult human Sertoli cells assumed similar morphological features, stable global gene expression profiles and numerous proteins, and activation of AKT and SMAD1/5 during long-period culture.

**Conclusions:**

This study demonstrates that adult human Sertoli cells can be cultured for a long period and expanded with remarkable increase of cell numbers whilst maintaining their primary morphology, phenotype and signaling pathways. This study could provide adequate human Sertoli cells for reproductive and regenerative medicine.

**Electronic supplementary material:**

The online version of this article (doi:10.1186/s12964-015-0101-2) contains supplementary material, which is available to authorized users.

## Introduction

Spermatogenesis is an elaborate process by which spermatogonial stem cells (SSCs) self-renew and differentiate into mature spermatozoa in mammalian testis. The microenvironment or niche is required for normal spermatogenesis. The niche comprises mainly Sertoli cells, cytokines and growth factors produced by Sertoli cells, peritubular myoid cells and blood vessels. As the key component of the niche of mammalian testis, Sertoli cells play essential roles in controlling spermatogenesis [1]. Sertoli cells are also called “mother cells” or “nurse cells” for male germ cells, reflecting a unique role for these cells in male germ cell development and maturation. Sertoli cells stretch from the basement membrane to the lumen within the seminiferous tubules, and they are in intimate contact with male germ cells and provide structural, immunological, and nutritional support to these cells [1].

Studies on Sertoli cells are of usual significance due to the following three aspects. First of all, Sertoli cells are a major orchestrator of the testis during sex differentiation and male germ cell development, as evidenced by the fact that they express Sry [2] and initiate the cascades of genes which regulate the formation of seminiferous cord and sex-specific vascular development [3-5]. Sertoli cells secret a number of growth factors and cytokines to control the self-renewal and differentiation of SSCs, meiosis of spermatocytes, and transformation of round spermatids to spermatozoa [6]. For instance, stem cell factor (SCF, also known as KIT ligand and steel factor) and bone morphogenic protein 4 (BMP4) secreted by Sertoli cells have been shown to induce the differentiation of SSCs [7-10]. Glial cell line-derived neurotrophic factor (GDNF), another critical growth factor produced by Sertoli cells, promotes the survival and self-renewal of mouse SSCs [11-16], since mice overexpressing GDNF have an accumulation of undifferentiated spermatogonia [13,17]. We have recently revealed distinct expression of SCF, BMP4, and GDNF in human Sertoli cells between non-obstructive azoospermia (NOA) patients with impaired spermatogenesis and obstructive azoospermia (OA) patients with normal spermatogenesis [18], suggesting that there might exist an association of these factors secreted by Sertoli cells and male infertility. Sertoli cells, serving as feeder cells, have been applied in the expansion and differentiation of SSCs in vitro [19-22]. Secondly, Sertoli cells are an essential component of testicular blood-testis-barrier (BTB) that is composed of Sertoli cell tight junctions, the body of the Sertoli cell, and peritubular cells [23-25]. The BTB has been regarded as the main defense against the invading immune cells which may attack immunogenic germ cells. Since most of male germ cells develop after the immune system is fully functional, they are autoimmunogenic [26]. Sertoli cells are considered as immunoprivileged cells, and they have been regarded as a major component of the defense against the immune system by expressing several immuno-regulatory factors, e.g., Fas ligand (FasL), transforming growth factor B1 (TGFB1) and clusterin [27-32]. Moreover, immune modulation by Sertoli cells is not restricted to the testes because Sertoli cells also survive when transplanted to ectopic sites [33-42]. Thirdly, Sertoli cells have been reprogrammed to generating the induced pluripotent stem (iPS) cells [43], and notably, Sertoli cells can be directly converted into morphologic, phenotypic, and functional neural stem cells [44]. Sertoli cells have been engineered to secrete the necessary proteins capable of restoring normal cell and metabolic function [45], indicating extreme promise of Sertoli cells to be exploited in cell-based therapy. Thus, Sertoli cells may have important implications for disease modeling and regenerative medicine.

Adult Sertoli cells have previously been considered the terminally differentiated cells with a fixed and unmodifiable population after puberty. Nevertheless, this concept has recently been challenged [46], because Sertoli cells from adult hamsters can regain proliferative ability after being administration of follicle-stimulating hormone and they have the proliferation potential [47]. Since the number of adult human Sertoli cells is limited, it is essential to culture these cells for a long period and expand them for their use in basic research and clinic applications. However, the studies of human Sertoli cells are restricted, due to the limited access to human testis tissues. In this study, we first isolated and purified adult human Sertoli cells from OA patients with normal spermatogenesis. We identified adult human Sertoli cells using numerous markers for Sertoli cells and then cultivated them for a long period, with a remarkable increase of cell numbers. We further compared morphology, phenotypic characteristics, and AKT and SMAD1/5 activation of adult human Sertoli cells at different passages. Significantly, we found that adult human Sertoli cells assumed similar morphological features and they had stable global gene expression profiling and a number of proteins, and AKT and SMAD1/5 activation during long-term cultivation. Considered together, we have demonstrated that adult human Sertoli cells can be cultured for long term and expanded while maintaining their primary morphology, phenotype, and signaling pathway activation, which underscores their promising applications in both reproductive and regenerative medicine.

## Materials and methods

### Procurement of testicular biopsies from OA patients with normal spermatogenesis

Testicular biopsies were obtained from 50 OA patients (age ranging from 22 to 35 years old) who underwent microdissection and testicular sperm extraction (MD-TEST) at Ren Ji Hospital affiliated to Shanghai Jiao Tong University School of Medicine. All patients with OA were caused by inflammation and vasoligation but not by congenital absence of the vas deferens (CBAVD) or other diseases including cancer. This study was approved by the Institutional Ethical Review Committee of Ren Ji Hospital (license number of ethics statement: 2012–01), Shanghai Jiao Tong University School of Medicine, and the consents for testicular biopsies were obtained from the donors for research only.

### Histological examination

Testicular biopsies from OA patients were fixed in Bouin’s fixative overnight, embedded in paraffin and sectioned at 5 μm thickness. The sections were stained with hematoxylin and eosin (H&E) and observed to check spermatogenesis status under a microscope.

### Isolation and identification of human Sertoli cells from OA patients

Testicular biopsies obtained from OA patients were washed three times aseptically in Dulbecco modified Eagle medium (DMEM) (Gibco) containing antibiotic with penicillin and streptomycin (Gibco). Human seminiferous tubules were isolated from testis biopsies by the first enzymatic digestion comprising 2 mg/ml collagenase IV (Gibco) and 1 μg/μl DNase I (Gibco) in 34°C water bath for 15 min pursuant to the procedure as described previously [48]. Sertoli cells and human male germ cells were obtained from seminiferous tubules using a second enzymatic digestion with 4 mg/ml collagenase IV, 2.5 mg/ml hyaluronidase (Sigma), 2 mg/ml trypsin (Sigma) and 1 μg/μl DNase I and followed by differential plating according to the procedure as we previously described [48]. For differential plating, cell suspension was seeded into culture plates in DMEM/F-12 supplemented with 10% fetal bovine serum (FBS) (Gibco) and incubated at 34°C in 5% CO_2_ overnight. After incubation, the medium containing male germ cells were removed, and Sertoli cells attached to culture plates. The viability of freshly isolated human Sertoli cells was assessed by exclusion of trypan blue staining. Human Sertoli cells were identified by immunostaining with antibodies against GATA4, WT1, vimentin (VIM), GDNF, BMP4, SCF, and PCNA (proliferating cell nuclear antigen) as described below.

### Long-term culture of human Sertoli cells from OA patients

Human Sertoli cells were cultured with DMEM/F12 supplemented 10% heat-inactivated FBS (Gibco), 2 mM L-glutamine (Invitrogen), and 100 unit/ml penicillin and streptomycin (Gibco). The cells were passaged when cell confluence reached 80%, and they were maintained at 34°C in a humidified 5% CO_2_ incubator and cultivated for 2 months. The proliferation potentials of human Sertoli cells at different passages were detected by cell counting using the cytometer.

### RNA extraction and reverse transcription-polymerase chain reaction (RT-PCR)

Total RNA was extracted from freshly isolated and passaged human Sertoli cells using the Trizol reagent (Invitrogen), and the quality and concentrations of total RNA were measured by Nanodrop. Reverse transcription (RT) was performed using the First Strand cDNA Synthesis Kit (Thermo Scientific), and PCR of the cDNA was carried out according to the protocol as described previously [49]. The primers of the chosen genes, including *GATA1* (GATA binding protein 1), *GATA4* (GATA binding protein 4), *WT1* (Wilms tumor 1), *GDNF*, *BMP4*, *SCF*, *FGF2* (fibroblast growth factor 2), *EGF* (epithelial growth factor), *FSHR* (follicle-stimulating hormone receptor), *AR* (androgen receptor), *ABP* (androgen binding protein, also known as sex hormone-binding globulin, SHBG), and *ACTB* (actin beta), were designed and listed in Table 1. The PCR reaction started at 94°C for 2 min and was performed as follows: denaturation at 94°C for 30 sec, annealing at 55-60°C for 45 sec as listed in Table 1, and elongation at 72°C for 45 sec. After 35 cycles, the samples were incubated for an additional 5 min at 72°C. PCR products were separated by electrophoresis on 2% agarose gel and visualized with ethidium bromide. Images were recorded and band intensities were analyzed using chemiluminescence (Chemi-Doc XRS, Bio-Rad) [18]. RNA without reverse transcriptase enzyme but with PCR of *ACTB* primers served as negative controls. The integrated density values (IDV) of target gene products were quantified relatively by comparing with the expression of housekeeper gene *ACTB*.

**Table 1:** **The sequences of gene primers used for RT-PCR**

**Gene**	**Primer sequence**		**Product size (bp)**	**Tm(°C)**
*WT1*	Forward	TGACTCTCCACTCCTCCTCAC	170	60
	Reverse	ACCAACTCTTCCAGGCACAC		
*GATA4*	Forward	GCCTCCTCTGCCTGGTAAT	210	60
	Reverse	CAGTCCCATCAGCGTGTAAA		
*GATA1*	Forward	GAAACCGCAAGGCATCTG	140	60
	Reverse	CCCAGCCACCACCATAAAG		
*GDNF*	Forward	TGAAACCAAGGAGGAACT	180	58
	Reverse	CACTCACCAGCCTTCTATT		
*BMP4*	Forward	TTTGTTCAAGATTGGCTGTC	324	60
	Reverse	AGATCCCGCATGTAGTCC		
*SCF*	Forward	GTCATTGTTGGATAAGCGAGAT	457	60
	Reverse	ATGGCTGCCCAGTGTAGG		
*FGF2*	Forward	TCCTTTCTCCCTCGTTTCTTC	195	55
	Reverse	GATGTTTCCCTCCAATGTTTC		
*EGF*	Forward	CTGATATAGATGAATGTGAGATGGG	190	55
	Reverse	GTGGAGTAGAGTCAAGACAG		
*FSHR*	Forward	TCTGCTGGTTCTGTTTCA	215	58
	Reverse	CATTCCTTGGATGGGTGT		
*AR*	Forward	CCTTCACCAATGTCAACTCC	197	60
	Reverse	CCACTGGAATAATGCTGAAGAG		
*ABP*	Forward	GGCTTTACCAGGAGAAGAC	150	60
	Reverse	CATGGCTTCTGTTCAGGG		
*ACTB*	Forward	CGCACCACTGGCATTGTCAT	253	55
	Reverse	TTCTCCTTGATGTCACGCAC		

### Immunocytochemistry

For immunocytochemical staining, the freshly isolated and cultured human Sertoli cells as well as human male germ cells were fixed with 4% paraformaldehyde (PFA) for 30 min, washed three times with cold phosphate-buffered saline (PBS) and permeabilized in 0.4% triton-X 100 (Sigma) for 5 min. After washing with PBS, the cells were blocked in 2% BSA for 30 min and followed by incubation with primary antibodies, including anti-GATA4 (Santa Cruz), WT1 (Santa Cruz), GDNF (Santa Cruz), anti-SCF (Sigma), anti-BMP4 (Abcam), anti- VIM (Santa Cruz), anti-PCNA (Sigma), anti-SMA (smooth muscle alpha actin, Abcam) and CYP11A1 (cholesterol side-chain cleavage enzyme, Abcam) at a dilution with 1:200 overnight at 4°C. Isotype IgGs for the first antibody were used as the negative controls. After extensive washes with PBS for 30 min, the cells were incubated with the secondary antibody IgG (Sigma) conjugated with fluorescein isothiocyanate (FITC) or rhodamine at a 1:200 dilution for 1 hour at room temperature. DAPI (4,6-diamidino-2-phenylindole) was used to label the nuclei, and the cells were observed for epifluorescence under a fluorescence microscope (Leica).

### Microarray analysis

Total RNA was obtained from human Sertoli cells at passage one (P1), passage five (P5) and passage ten (P10) using Trizol reagent (Invitrogen). The concentrations and purity of total RNA were evaluated by measuring the 260/280 nm ratio, and the integrity of total RNA was assessed by 1.0% denaturing agarose gel electrophoresis. Five micrograms of total RNA were reverse-transcribed into single strand cDNA that was subsequently converted to double strand cDNA. The purified double strand cDNA served as a template to synthesize biotin-labeled cRNA using the GeneChip2 WT Terminal Labeling Kit (Affymetrix), and the biotin-labeled cRNA was purified using the GeneChip Sample Cleanup Module kit (Affymetrix) and fragmented to 35–200 bases. The fragmented cRNA was hybridized to Affymetrix GeneChip Human Gene 1.0 ST Arrays and stained using the GeneChip2 Hybridization, Wash, and Stain Kit (Affymetrix). The arrays were scanned with a GeneChip2 Scanner 3000 7G (Affymetrix), and raw data were analyzed using the GeneSpringGX software.

### Western blots

The cultured human Sertoli cells at different passages were lysed with RIPA buffer (Santa Cruz) for 30 min on ice. After 30 min lysis on ice, cell lysates were cleared by centrifugation at 12,000 g, and the concentration of protein was measured by BCA kit (Dingguo Company, China). Thirty micrograms of cell lysate from each sample were used for SDS-PAGE (Bio-Rad Laboratories, Richmond, CA), and Western blots were performed according to the protocol as described previously [11]. The chosen antibodies included anti-SCF (Sigma), anti-GDNF (Santa Cruz), anti-BMP4 (Abcam), anti-PCNA (Santa Cruz), anti-phos-AKT (Cell Signaling), and anti-phos-SMAD1/5 (Cell Signaling). After extensive washes with PBS, the blots were detected by chemiluminescence (Chemi-Doc XRS, Bio-Rad), and the integrated density values (IDV) was calculated by comparing the signals of target proteins with that of housekeeper ACTB (Santa Cruz).

### Statistical analysis

All the values were presented as mean ± SEM, and statistically significant differences (p < 0.05) between different passages of human Sertoli cells were determined using the analysis of variance (ANOVA) and a 2-tailed t-test. The band intensities of Western blots were quantified by ImageJ software (NIH).

## Results

### Isolation of human Sertoli cells from OA patients

**Figure 1:**
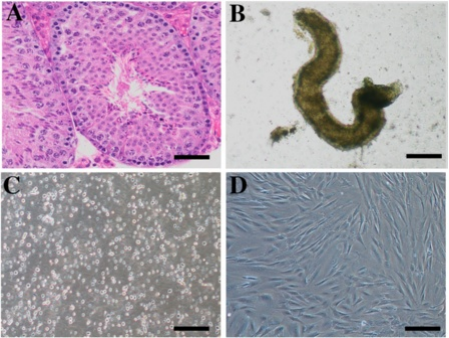
**Morphology of the testes from OA patients and the isolation of human Sertoli cells from patients with OA. (A)** H&E staining revealed the morphology of testicular tissues from OA patients. **(B)** Seminiferous tubules were obtained after the first enzymatic digestion with collagenase IV and DNase I. **(C)** The mixture of male germ cells and Sertoli cells was isolated from seminiferous tubules using the second enzymatic digestion. **(D)** Sertoli cells remained in the culture plates after differential plating with the removal of male germ cells. Scale bars in **A-C** = 100 μm; scale bar in **D** = 50 μm.

Histological analyses of testicular tissues revealed that 50 OA patients we used in this study had normal spermatogenesis (Figure 1A). Human Sertoli cells were isolated from OA patients by a two-step enzymatic digestion and followed by differential plating as previously described [48]. In brief, human seminiferous tubules (Figure 1B) were obtained from the testis tissues using the first enzymatic digestion and extensive washes using PBS to remove potential contamination of myoid cells. Male germ cells and Sertoli cells (Figure 1C) were obtained using a second enzymatic digestion and they were cultured with DMEM/F12 and 10% FBS overnight for differential plating. Human Sertoli cells (Figure 1D) attached to the plates for the subsequent culture. The viability of freshly isolated cells was over 98%, as assayed by trypan blue exclusion (data not shown).

### Identification of human Sertoli cells from OA patients

**Figure 2:**
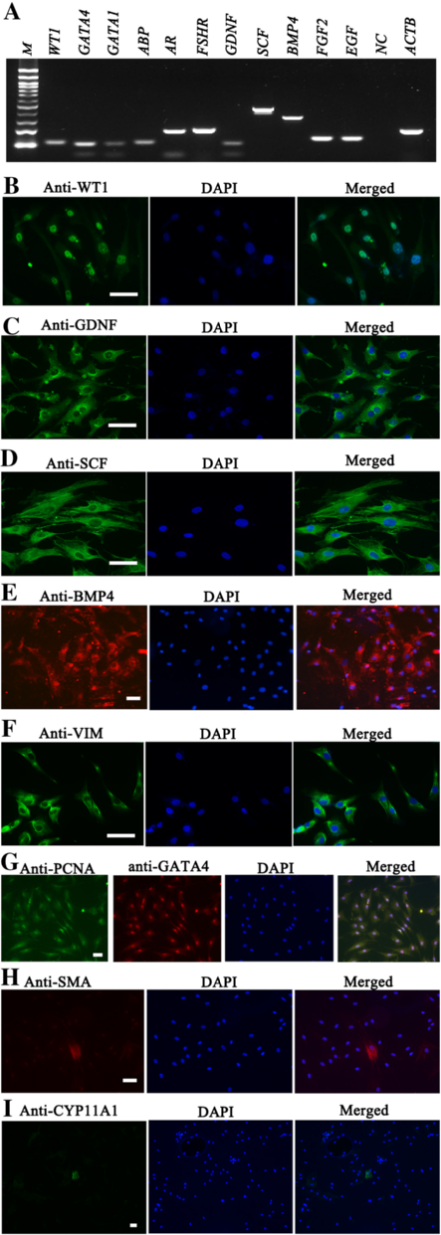
**Gene and protein characterization of the freshly isolated human Sertoli cells. (A)** RT-PCR showed the expression of numerous genes, including *WT1*, *GATA4*, *GATA1*, *GDNF*, *BMP4*, *SCF*, *FGF2*, *EGF*, *FHSR*, *AR* and *ABP. ACTB* was used as a loading control, and RNA without reverse transcriptase enzyme but with PCR of *ACTB* primers was used as a negative control (NC). **(B-I)** Immunofluorescence revealed the expression of WT1 **(B)**, GDNF **(C)**, SCF **(D)**, BMP4 **(E)**, VIM **(F)**, PCNA and GATA4 **(G)**, SMA **(H)**, and CYP11A1 **(I)** in isolated human Sertoli cells. Scale bars in **B**, **C**, **D**, **F**, **H** =50 μm; scale bars in **E**, **G**, **I** =20 μm.

The freshly isolated human Sertoli cells were identified using a number of markers of Sertoli cells. RT-PCR showed that the transcripts of *WT1*, *GATA4*, *GATA1*, *GDNF*, *BMP4*, *SCF*, *FGF2*, *EGF*, *FHSR*, *AR* and *ABP* were expressed in the isolated Sertoli cells (Figure 2A). Immunocytochemistry further revealed that primary human Sertoli cells were positive for WT1 (Figure 2B), GDNF (Figure 2C), SCF (Figure 2D), BMP4 (Figure 2E), VIM (Figure 2F), and PCNA and GATA4 (Figure 2G). No positive staining was seen when primary antibodies were replaced with isotype rabbit or goat IgGs (Additional file 1: Figure S1) or in human male germ cells with these antibodies (Additional file 2: Figure S2), confirming the specific expression of these proteins in freshly isolated human Sertoli cells. The purity of isolated Sertoli cells was more than 95% as showed by our immunostaining results that less than 5% of the cells were positive for antibodies against SMA (Figure 2H) or CYP11A1 (Figure 2I), markers for myoid cells and Leydig cells, respectively. To assess the proliferation ability of human Sertoli cells, PCNA expression was measured and almost of the cells were observed to be positive for both PCNA and GATA4 (Figure 2G), reflecting that human Sertoli cells have a high level of proliferative potential.

### Long-term culture of human Sertoli cells

**Figure 3:**
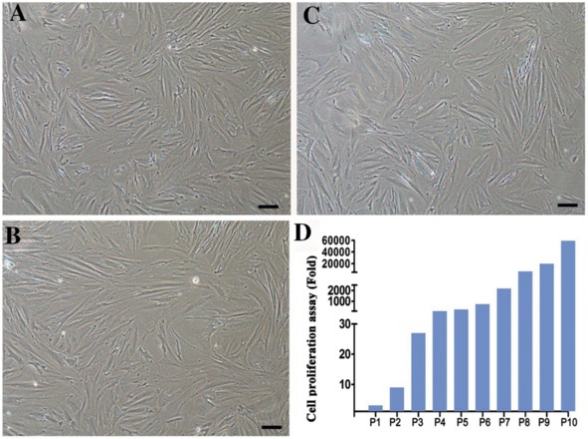
**Morphology and proliferation assay of human Sertoli cells in culture. (A-C)** Phase-contrast microscope displayed the morphological characteristics of human Sertoli cells after culture for P1 **(A)**, P5 **(B)**, and P10 **(C)**. Scale bars in **A-C** = 50 μm. **(D)** Cell proliferation assay showed the proliferative ability of human Sertoli cells when cultured for 2 months.

When human Sertoli cells reached 80% of confluence, they were passaged by the ratio 1:3. Adult human Sertoli cells could be passaged every 4 to 5 days until 2 months with 10 passages. We compared the morphological features of human Sertoli cells at passage one (P1), passage five (P5) and passage ten (P10). Under the phrase-contrast microscope, human Sertoli cells at P1, P5 and P10 assumed similar morphology, as evidenced by the observations that they had a large cell body, a branching cytoplasm, and irregular nuclei (Figure 3A-C). Cell proliferation assay showed that adult human Sertoli cells could be expanded with a remarkable increase of cell number by 59,049 folds after culture for 10 passages (Figure 3D), which further suggests that human Sertoli cells possess a significant proliferation ability.

### Global gene expression profiles of human Sertoli cells at different passages

**Figure 4:**
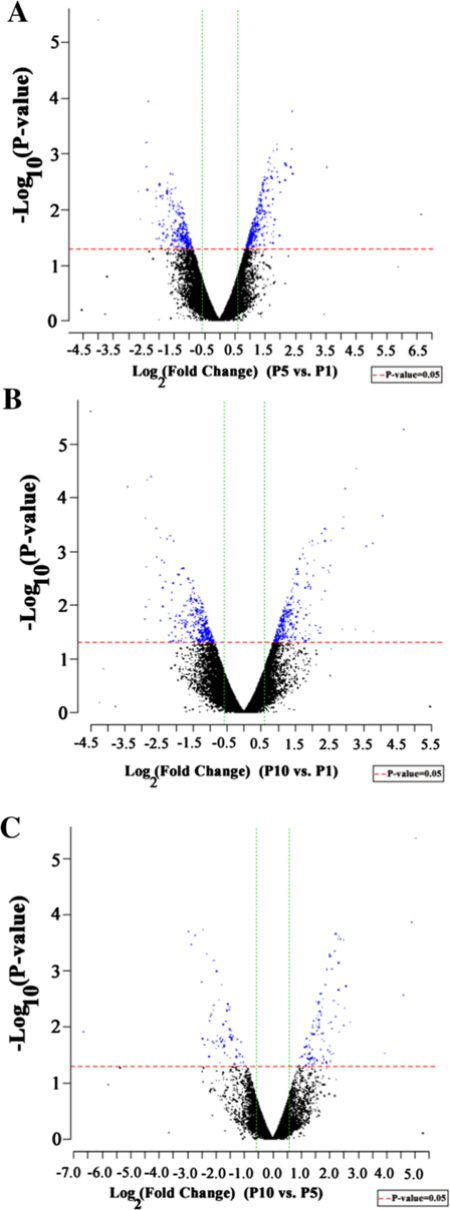
**Microarray analysis revealed global gene expression patterns in human Sertoli cells at different passages. (A-C)** Scatter plots displayed the large scale gene expression profiles in human Sertoli cells at P5/P1 **(A)**, P10/P1 **(B)**, and P10/P5 **(C)**. Each dot presented one gene or the expressed sequence tags (EST).

We next compared global gene expression profiling of human Sertoli cells at P1, P5 and P10 using microarray analysis. To this end, total RNA was extracted from human Sertoli cells at different passages, and gel imaging and electropherograms by Agilent bioanalyzer assays displayed that RNA was of great quality (Additional file 3: Figure S3A-E). Microarray analysis revealed that totally 31,743 genes and the expressed sequence tags (EST) were detected in human Sertoli cells (Figure 4A-C). There were 597 (1.88%), 663 (2.09%), and 177 (0.56%) differentially expressed genes (up-regulated or down-regulated with 2.0 folds or more) in Sertoli cells at P5/P1 (Figure 4A, Table 2), P10/P1 (Figure 4B, Table 2), and P10/P5 (Figure 4C, Table 2), respectively. Meanwhile, a number of important genes for Sertoli cells, including *WT1, GATA4, GATA1, GDNF, BMP4, KITLG* (also called *SCF*), *FGF2*, *EGF*, *FSHR*, *AR* and *ABP*, were stably expressed in human Sertoli cells after culture for P1, P5 and P10, since their expression was changed with less than 2.0 folds (Table 3).

**Table 2:** **The number of the differentially expressed genes in human Sertoli cells at P5/P1, P10/P1 and P10/P5 by microarray analysis**

**Group**	**Up-regulated genes**	**Down-regulated genes**	**Percentage**
P5/P1	307	290	1.88%
P10/P1	324	339	2.09%
P10/P5	106	71	0.56%

**Table 3:** **The expression of numerous genes in human Sertoli cells at P5/P1, P10/P1 and P10/P5 by microarray analysis**

**Gene symbol**	**Description**	**Normalized Intensity**			**log**_**2**_ **(Ratio)**		
		**P1**	**P10**	**P5**	**P10/P1**	**P5/P1**	**P10/P5**
*WT1*	Wilms tumor 1	219.234818	161.693756	198.794189	−0.774764	−0.36631	−0.420787
*GATA4*	GATA binding protein 4	3865.29419	4788.86475	5275.979	0.295279	0.40343	−0.128533
*GATA1*	GATA binding protein 1	1037.77979	1873.12756	1690.21729	0.715539	0.529079	0.104663
*KITLG*	KIT ligand	170.060654	236.24087	244.52618	0.13905	0.297491	−0.169023
*GDNF*	glial cell derived neurotrophic factor	235.626205	198.442337	173.594925	−0.584025	−0.668577	0.071242
*BMP4*	bone morphogenetic protein 4	13598.7051	17179.4355	19054.377	0.351068	0.44625	−0.114685
*FGF2*	fibroblast growth factor 2 (basic)	10512.002	10258.1035	10363.4307	−0.045128	−0.060692	−0.008951
*EGF*	epidermal growth factor	210.014664	313.937866	237.059738	0.238724	−0.052155	0.285537
*FSHR*	follicle stimulating hormone receptor	14.342464	38.848499	21.46604	1.437565932	0.5817631	0.8558028
*AR*	androgen receptor	2127.80713	1999.12268	2071.93945	−0.160308	−0.174322	−0.08023
*SHBG/ABP*	sex hormone-binding globulin	389.295471	413.684021	454.52005	−0.254276	−0.036372	−0.231509

**Figure 5:**
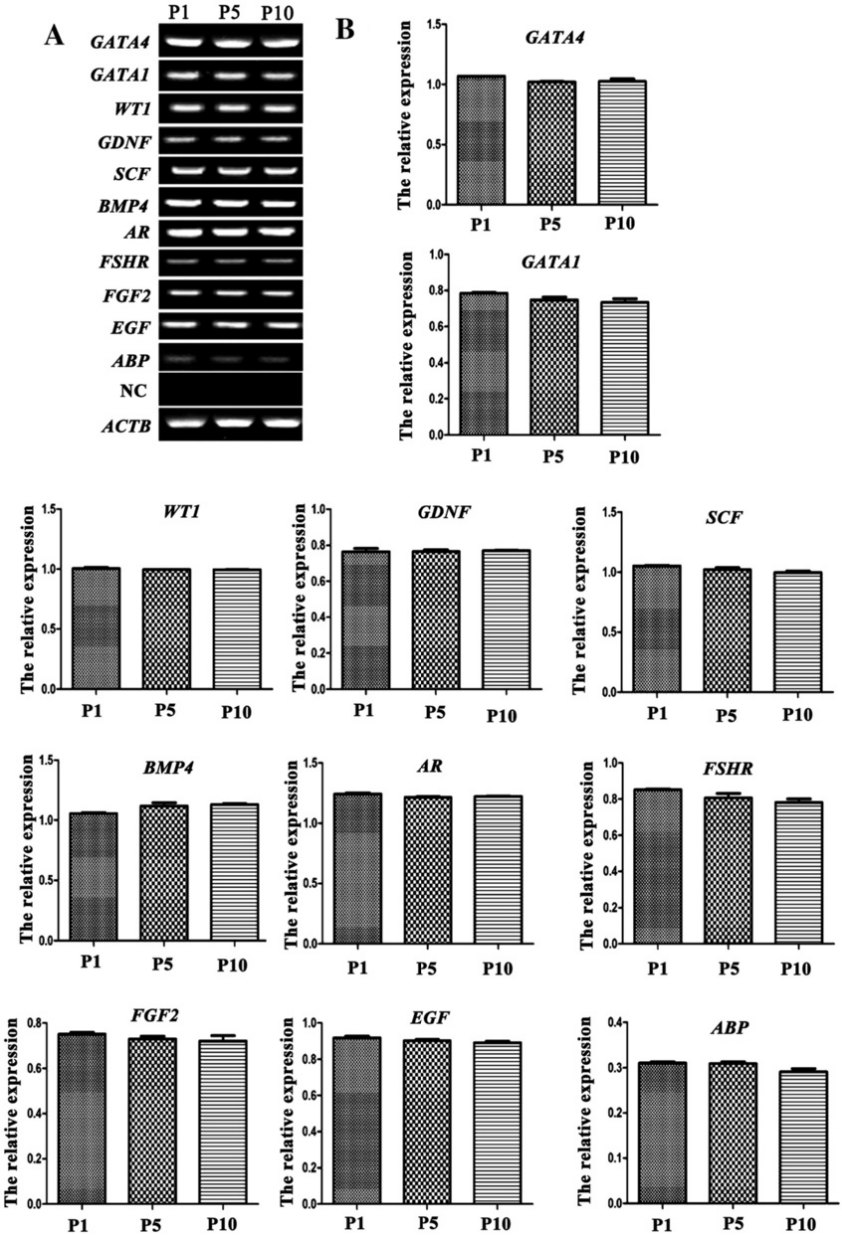
**The expression of** ***WT1, GATA4, GATA1, GDNF, BMP4, KITLG*** **,** ***FGF2*** **,** ***EGF*** **,** ***FSHR*** **,** ***AR*** **and** ***ABP*** **in human Sertoli cells after culture for P1, P5 and P10. (A, B)** RT-PCR showed the transcripts of *WT1, GATA4, GATA1, GDNF, BMP4, KITLG*, *FGF2*, *EGF*, *FSHR*, *AR* and *ABP* in human Sertoli cells at P1, P5 and P10. *ACTB* was used as a loading control, and RNA without reverse transcriptase enzyme but with PCR of *ACTB* served as a negative control (NC). There was no significant difference (P >0.05) in the gene expression levels of *WT1, GATA4, GATA1, GDNF, BMP4, KITLG*, *FGF2*, *EGF*, *FSHR*, *AR* and *ABP* in human Sertoli cells after culture for P1, P5 and P10.

To confirm the results of microarray analysis, RT-PCR was performed to check the expression of *WT1, GATA4, GATA1, GDNF, BMP4, KITLG* (*SCF*), *FGF2*, *EGF*, *FSHR*, *AR*, and *ABP*. Notably, there was no significantly statistical difference between the expression of these genes mentioned above in human Sertoli cells at P1, P5, P10 (Figure 5A and B), which were consistent with the data of our mRNA microarray.

### Stable expression of numerous proteins in human Sertoli cells at P1, P5, and P10

**Figure 6:**
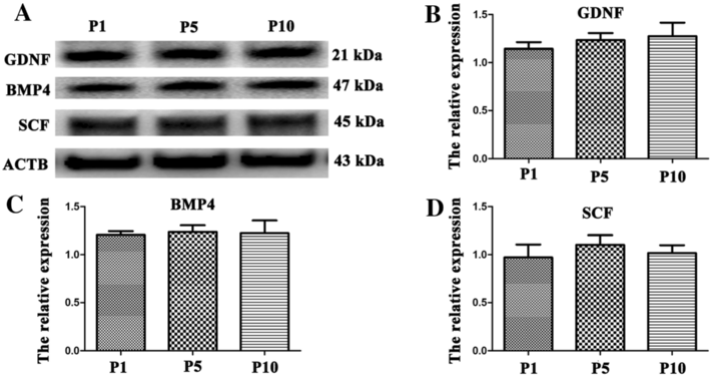
**Protein expression of GDNF, SCF and BMP4 in human Sertoli cells at different passages. (A-D)** Western blots showed the expression of GDNF, SCF and BMP4 in human Sertoli cells at P1, P5 and P10. ACTB was used as the control of loading proteins.

Due to the important function of GDNF, SCF and BMP4 in the regulation of spermatogenesis, we further examined the protein expression of these growth factors in human Sertoli cells at P1, P5, and P10. Western blots revealed that GDNF, SCF and BMP4 proteins were stably expressed in human Sertoli cells when they were cultured for 1, 5, and 10 passages (Figure 6A-D).

Immunofluorescence further showed the expression of WT1, GDNF, SCF, BMP4, VIM, PCNA and GATA4 was retained at higher levels in human Sertoli cells after culture for 5 passages (Additional file 4: Figure S4) and 10 passages (Additional file 5: Figure S5), which was comparable to the expression of these proteins in freshly isolated human Sertoli cells (Figure 2). In contrast, SMA and CYP11A1 were detectable in very few human Sertoli cells after culture for 5 passages (Additional file 4: Figure S4) and 10 passages (Additional file 5: Figure S5).

### Expression of PCNA, phos-AKT and phos-SMAD1/5 in human Sertoli cells at different passages

**Figure 7:**
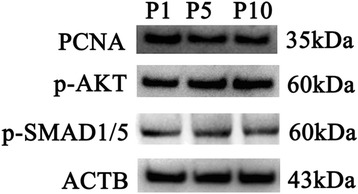
**Expression of PCNA, phos-AKT and phos-SMAD1/5 in human Sertoli cells after culture for 2 months.** Western blots displayed the expression of PCNA, phos-AKT, and phos-SMAD1/5 in human Sertoli cells at P1, P5, P10. ACTB served as the control of loading proteins.

We also compared the proliferation potential and signaling pathways of human Sertoli cells after culture for 1, 5, and 10 passages. Western blots showed that there was no difference in the expression of PCNA in human Sertoli cells at P1, P5, and P10 (Figure 7), and phos-AKT and phos-SMAD1/5 were expressed at similar levels in human Sertoli cells at P1, P5, and P10 (Figure 7). These data suggest that the activation of AKT and SMAD1/5 signaling pathways was maintained in human Sertoli cells during the long-period culture.

## Discussion

Sertoli cells are essential for spermatogenesis and testis development. The unique and appropriate regulation of the niche is required for normal spermatogenesis, and the integrity of niche relies on Sertoli cells [50]. Sertoli cells keep close contact with all male germ cells, including spermatogonia, spermatocytes, round spermatids and elongated spermatids [51], and they provide structural, immunological and nutritional support to each step of male germ cell development [52]. Sertoli cells could secret a number of growth factors and cytokines that are pivotal for spermatogenesis [50,53]. Therefore, it is of great significance to understand the biology of Sertoli cells, which could have great impact on male reproduction and on cell-based therapy in regenerative medicine. However, there are very few studies on human Sertoli cells because it is rather difficult to obtain human testis tissues. In this study, we have isolated and cultured adult human Sertoli cells for a long period with a significant increase of cell number, which makes it feasible for the wide application of Sertoli cells in both basic research and clinical use.

We obtained the biopsies of testis tissue from OA patients who underwent MD-TESE. The OA patients had integral and normal spermatogenesis as shown by our histological analyses of testicular tissues. Using the two-step enzymatic digestion and followed by differential plating, Sertoli cells were isolated with a high viability from OA patients. We found that numerous genes, including *WT1*, *GATA1*, *GATA4*, *GDNF*, *BMP4*, *SCF*, *FGF2*, *EGF*, *FHSR*, *AR* and *ABP*, were expressed in freshly isolated human Sertoli cells. The transcription factor *WT1* is expressed in Sertoli cells [54] and it is a marker of Sertoli cells [55]. *WT1* is critical for mouse spermatogenesis via the regulation of Sertoli cell polarity and it is associated with NOA [56], suggesting that WT1 mutation is one of the genetic causes of NOA in humans. Gata factors are important transcriptional regulators of Sertoli cell-specific gene expression [57,58], and Sertoli cells are known to express GATA 1, 4 and 6 [59,60]. Mutation in the GATA1 has been reported in cryptorchidism [61], implicating GATA1 deficiency is associated male reproductive disorder. GATA4 is expressed in Sertoli cells [62], and Gata4 conditionally knockout mice have testicular atrophy and loss of fertility with Sertoli cell vacuolation [63], suggesting that GATA4 is a key transcriptional regulator of Sertoli cells and spermatogenesis. GDNF is secreted by Sertoli cells [13,64]. Overexpression of GDNF stimulates mouse self-renewal of SSCs and GDNF-null allele results in Sertoli cell-only seminiferous tubules [13]. A large number of studies have showed that GDNF is essential for the self-renewal of mouse SSCs [1,15,65]. We have demonstrated that GDNF promotes mouse SSC proliferation by acting on c-Fos transcription through the Ras/Erk 1/2 pathway [11]. BMP4 is synthesized by Sertoli cells [10], and it exerts both mitogenic and differentiation effects [10]. SCF can induce mouse spermatogonia to differentiate into meiotic spermatocytes and eventually haploid round spermatids [8]. SCF is present in human Sertoli cells and its transcript is lower in testicular biopsies of patients with spermatogenic failure and in Sertoli cells of NOA patients [18,66,67]. Therefore, SCF may contribute to the deficiency in sterility. It has been demonstrated that SCF is essential for inducing the differentiation of SSCs [68]. FGF2 is secreted by Sertoli cells, Leydig cells and germ cells [69], and it is required for SSC self-renewal [48,70-74]. Moreover, FGF2 can strongly stimulate GDNF transcript to facilitate the division of SSCs [75,76]. EGF is crucial for spermatogenesis [75] and it stimulates the expansion of SSCs in vitro [48,72-74]. It has been shown that submandibular gland ablation leads to decreased circulating EGF and infertility, which could be reversed by EGF in mice [77], highlighting an important role of EGF in spermatogenesis. In addition, Sertoli cells exclusively possess receptor for FSH and they are the major targets of the ultimate hormonal signals to regulate spermatogenesis [78]. A number of independent studies have identified FSH-inducible genes (e.g., FSHR, ABP, GDNF, and SCF) in Sertoli cells that have direct effects in supporting spermatogenesis. It has recently been reported that at least 300 genes are regulated by FSH in Sertoli cells [79,80], reflecting a potential role of FSH/FSHR in controlling the fate of Sertoli cells. ABP is a testicular glycoprotein which binds androgens with high affinity and transports them to the epididymis [81-83]. ABP has been implicated in regulating androgen activity in the testis [84]. It is well established that androgen is essential for the integrity of spermatogenesis [85,86], and the biological function is mediated by AR present in Sertoli cells [78,87]. Nuclear immunoexpression of AR is a feature of mature Sertoli cells [55], and ablation of androgen signaling in Ar knock-out or in Sertoli cell-specific Ar knock-out mice results in complete sterility [85,88-90], suggesting that AR is essential for the maintenance of spermatogenesis. Our results showed that the above genes and proteins were present in the freshly isolated cells, implicating that these cells were human Sertoli cells in phenotypes. The purity of freshly isolated Sertoli cells was more than 95%, since our immunocytochemistry revealed that over 95% of these cells were positive for WT1, GATA4, GDNF, BMP4, SCF and VIM, hallmarks for Sertoli cells, but not for SMA or CYP11A1, markers for myoid and Leydig cells. Human Sertoli cells had typical morphology which was well maintained until 2 months of culture.

Adult Sertoli cells have previously been considered the terminally differentiated cells with a fixed and unmodifiable population after puberty [91-94]; however, there is evidence suggesting that Sertoli cells are not terminally differentiated and quiescent [46,47]. It has been shown that mouse and human adult Sertoli cells can be cultivated with 5% fetal calf serum (FCS) for 20 days with a proliferation ability [95]. Here we found that adult human Sertoli cells can be expanded and cultured for more than 2 months with a significant increase by 59,049-fold of cell numbers when they were 10% FBS, reflecting that 10% FBS seems to be better than 5% FCS for the proliferation of adult Sertoli cells. Almost all the cultured human Sertoli cells were positive of PCNA, a marker for proliferating cells [96], reflecting a strong the propagation ability of adult human Sertoli cells. To probe the effect of long-term culture on the global gene expression profiles, microarray analysis was performed in human Sertoli cells at different passages. Significantly, we revealed that 98.1%, 97.9, and 99.4% of genes maintained stable expression in human Sertoli cells at P5/P1, P10/P1, P10/P5, respectively. Owing to the unique and essential roles of human Sertoli cells in regulating the self-renewal and differentiation of SSCs, Sertoli cells have been exploited as feeder to culture SSCs and induce the differentiation of SSCs and other types of stem cells. The stable expression of genomic genes guarantees proper function of Sertoli cells as feeder cells and the application in other related studies. Meanwhile, numerous genes, including *WT1*, *GATA4*, *GATA1*, *GDNF*, *BMP4*, *SCF*, *FGF2*, *EGF*, *FHSR*, *AR* and *ABP*, essential factors produced by Sertoli cells, were stably expressed during the long term culture of Sertoli cells, suggesting that our culture system has little effect on the expression profile and biological functions of human Sertoli cells. The stability of these genes expression profile was confirmed by RT-PCR. We also ascertained the stable protein expression of certain critical growth factors, namely GDNF, SCF and BMP4 in Sertoli cells at P1, P5 and P10. Previously we found that the expression of GDNF, SCF and BMP4 was lower in Sertoli cells from NOA patients compared with OA patients [18], which implies that GDNF, SCF and BMP4 secreted by human Sertoli cells are essential for normal human spermatogenesis. Notably, we found that human Sertoli cells maintained high levels of proliferation and activation of AKT and SMAD1/5 during long-term culture.

In summary, we have demonstrated that adult human Sertoli cells can be cultured for a long term and expanded with a significant increase of cell numbers, while maintaining their primary morphology, stable global gene expression patterns and a number of proteins, and acting signaling pathways. This study underscores that it is reliable and feasible that cultured human Sertoli cells can be exploited in better understanding testicular dysgenesis syndrome, developing novel treatment for male reproductive disorders, and regenerative medicine.

## Additional files

Additional file 1: Figure S1. Negative controls for immunocytochemistry. (A) Primary antibody was replaced by goat serum IgG. (B) Primary antibody was replaced by rabbit serum IgG. Scale bars in A-B = 20 μm. Additional file 2: Figure S2. Negative controls for immunocytochemistry. (A-F) Immunofluorescence revealed the expression of WT1(A), GDNF(B), BMP4(D), SCF(E), and VIM (E) in human male germ cells. Scale bars in B-H =10 μm. Additional file 3: Figure S3. The quality evaluation of RNA used for microarray analysis. (A) Agarose gel electrophoresis showed the integrity of total RNA used for microarray analysis. (B-E) Electropherogram by Agilent bioanalyzer displayed total RNA of human Sertoli cells at P1 (B), P5 (C) and P10 (D), and RNA ladder (E). Additional file 4: Figure S4. Expression of a number of proteins after culture for 5 passages. Immunofluorescence showed the expression of WT1 (A), GDNF (B), SCF (C), BMP4 (D), VIM (E), PCNA and GATA4 (F), SMA(G), and CYP11A1 (H) in human Sertoli cells at passage 5. Scale bars in A, B, C, E,G =50 μm; scale bars in D, F, H =20 μm. Additional file 5: Figure S5. Expression of a number of proteins after culture for 10 passages. Immunofluorescence revealed the expression of WT1 (A), GDNF (B), SCF (C), BMP4 (D), VIM (E), PCNA and GATA4 (F), SMA(G), and CYP11A1 (H) in human Sertoli cells at passage 10. Scale bars in A, B, C, E, F, G, H = 50 μm; scale bar in D =20 μm.
