# Supplementary figures and images for: Long-term culture and significant expansion of human Sertoli cells whilst maintaining stable global phenotype and AKT and SMAD1/5 activation
Source: Cell Commun Signal. 2015 Mar 25;13:20. doi: 10.1186/s12964-015-0101-2 (PMC4380114; doi:10.1186/s12964-015-0101-2)

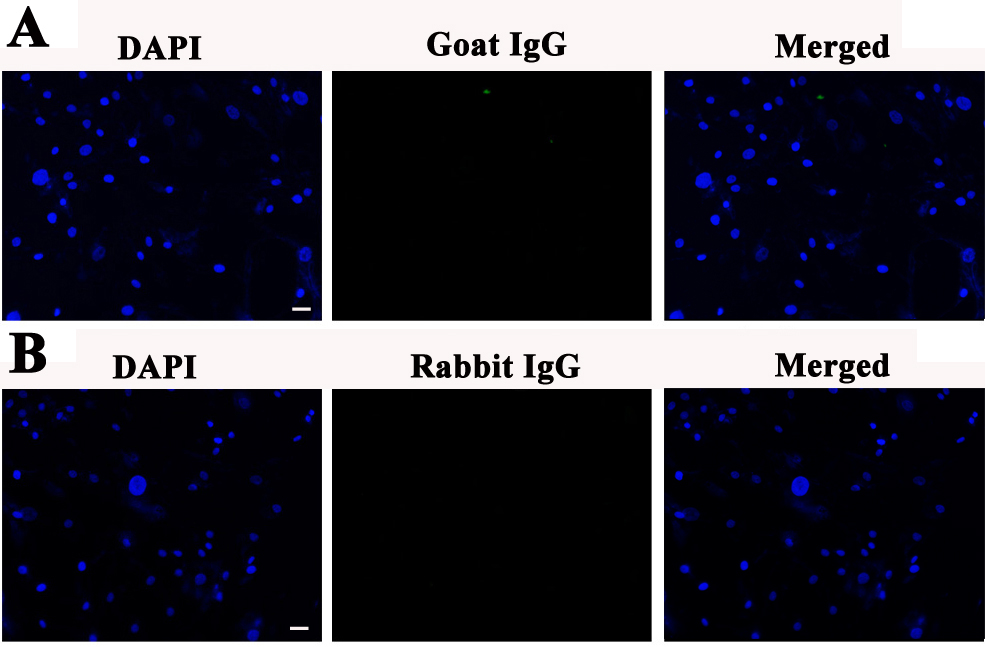

Supplement: Additional file 1: Figure S1. — Negative controls for immunocytochemistry. (A) Primary antibody was replaced by goat serum IgG. (B) Primary antibody was replaced by rabbit serum IgG. Scale bars in A-B = 20 μm. [file 12964_2015_101_MOESM1_ESM.jpeg]

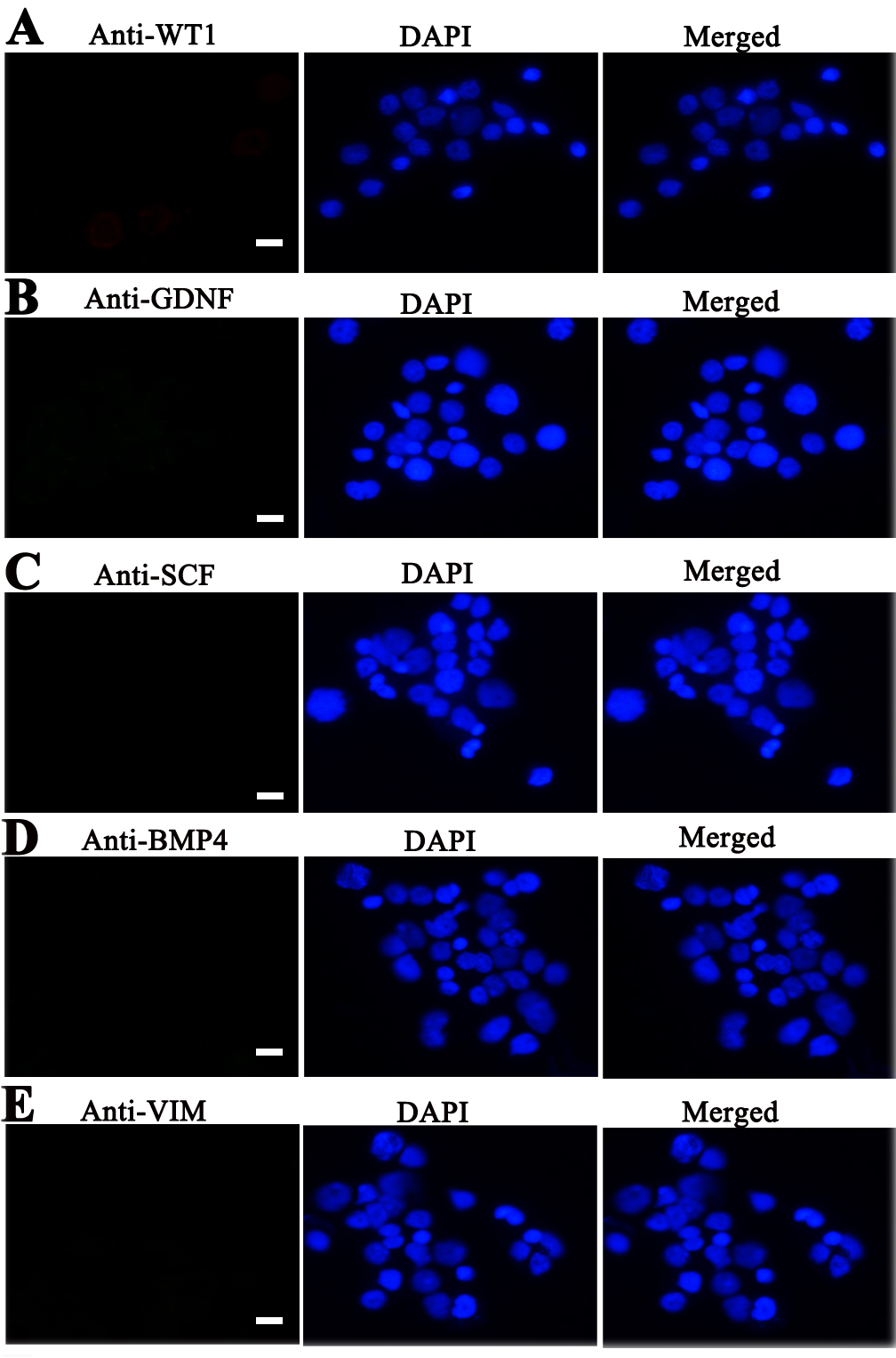

Supplement: Additional file 2: Figure S2. — Negative controls for immunocytochemistry. (A-F) Immunofluorescence revealed the expression of WT1(A), GDNF(B), BMP4(D), SCF(E), and VIM (E) in human male germ cells. Scale bars in B-H =10 μm. [file 12964_2015_101_MOESM2_ESM.jpeg]

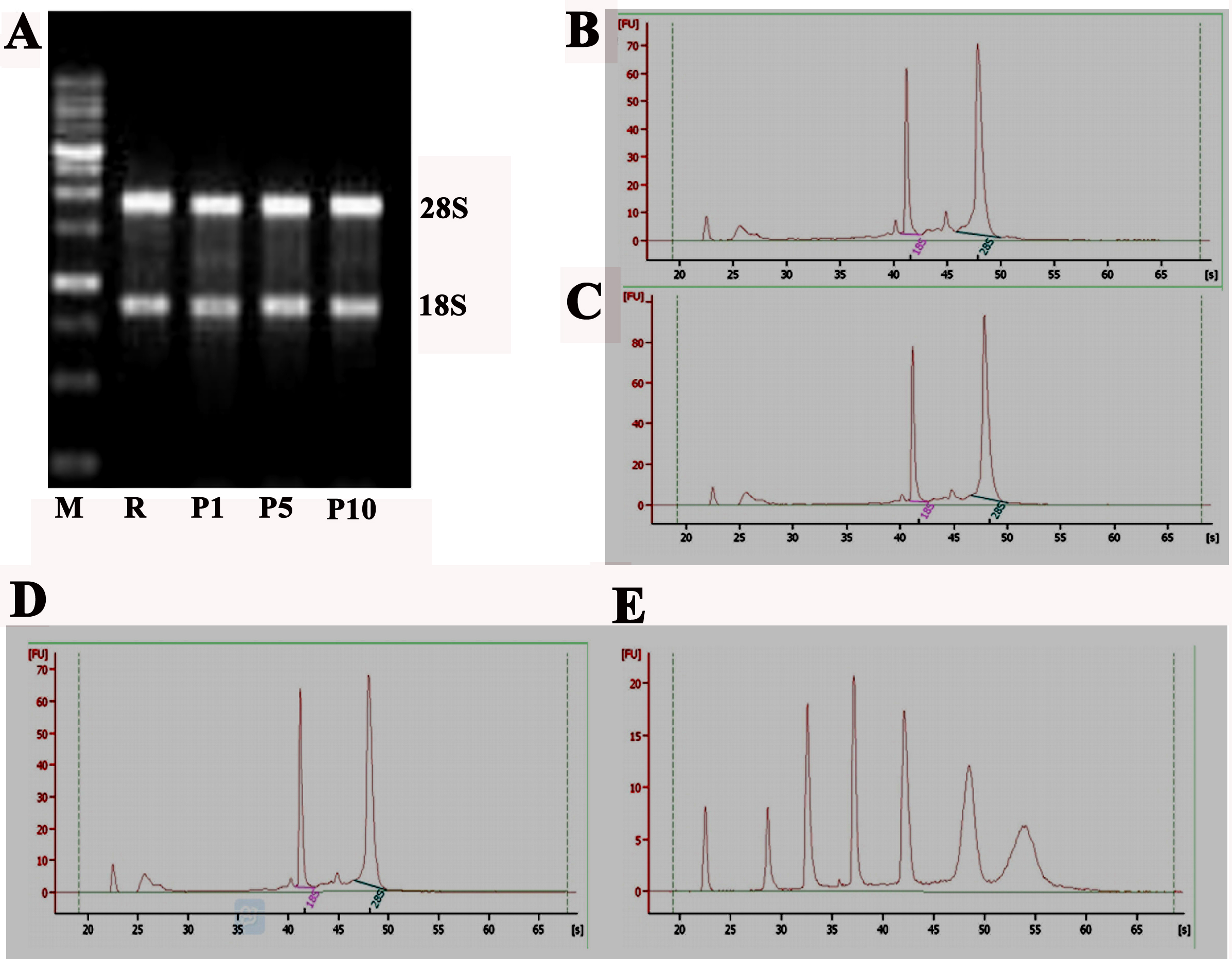

Supplement: Additional file 3: Figure S3. — The quality evaluation of RNA used for microarray analysis. (A) Agarose gel electrophoresis showed the integrity of total RNA used for microarray analysis. (B-E) Electropherogram by Agilent bioanalyzer displayed total RNA of human Sertoli cells at P1 (B), P5 (C) and P10 (D), and RNA ladder (E). [file 12964_2015_101_MOESM3_ESM.jpeg]

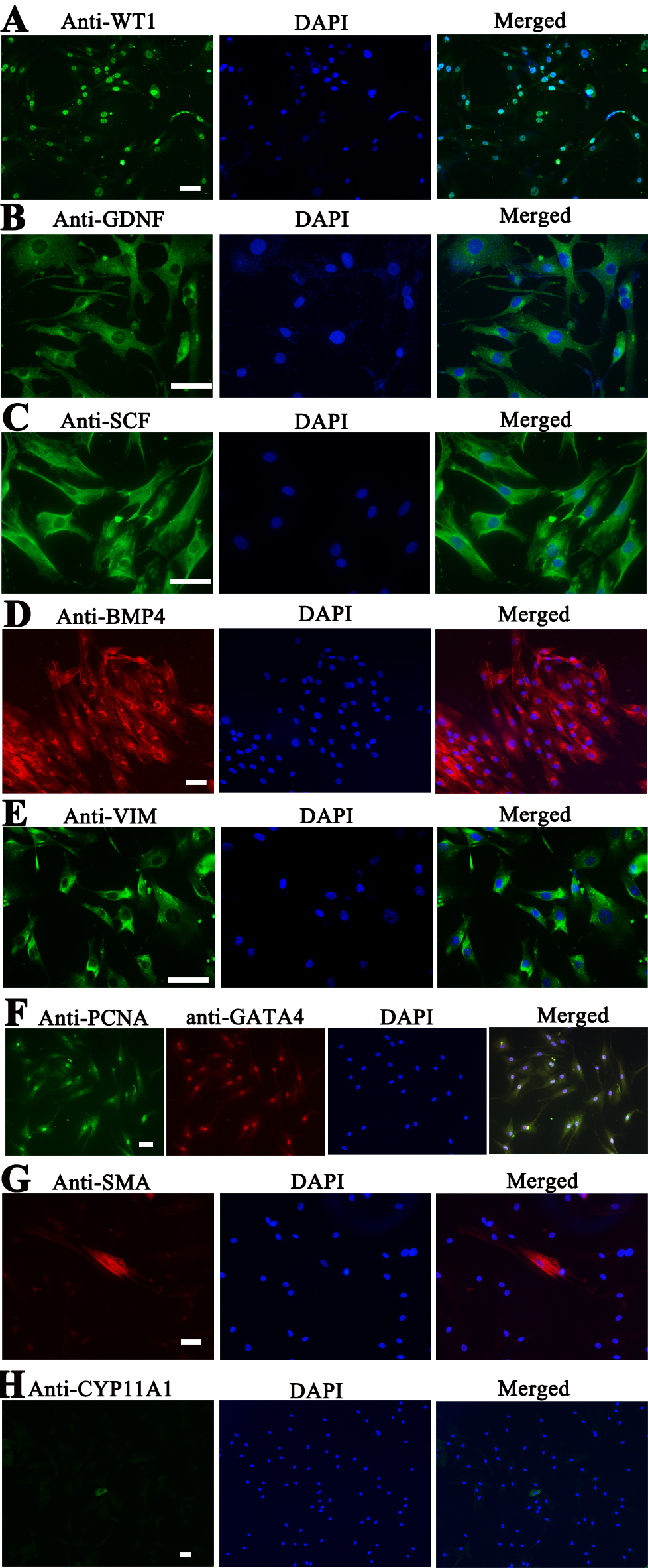

Supplement: Additional file 4: Figure S4. — Expression of a number of proteins after culture for 5 passages. Immunofluorescence showed the expression of WT1 (A), GDNF (B), SCF (C), BMP4 (D), VIM (E), PCNA and GATA4 (F), SMA(G), and CYP11A1 (H) in human Sertoli cells at passage 5. Scale bars in A, B, C, E,G =50 μm; scale bars in D, F, H =20 μm. [file 12964_2015_101_MOESM4_ESM.jpeg]

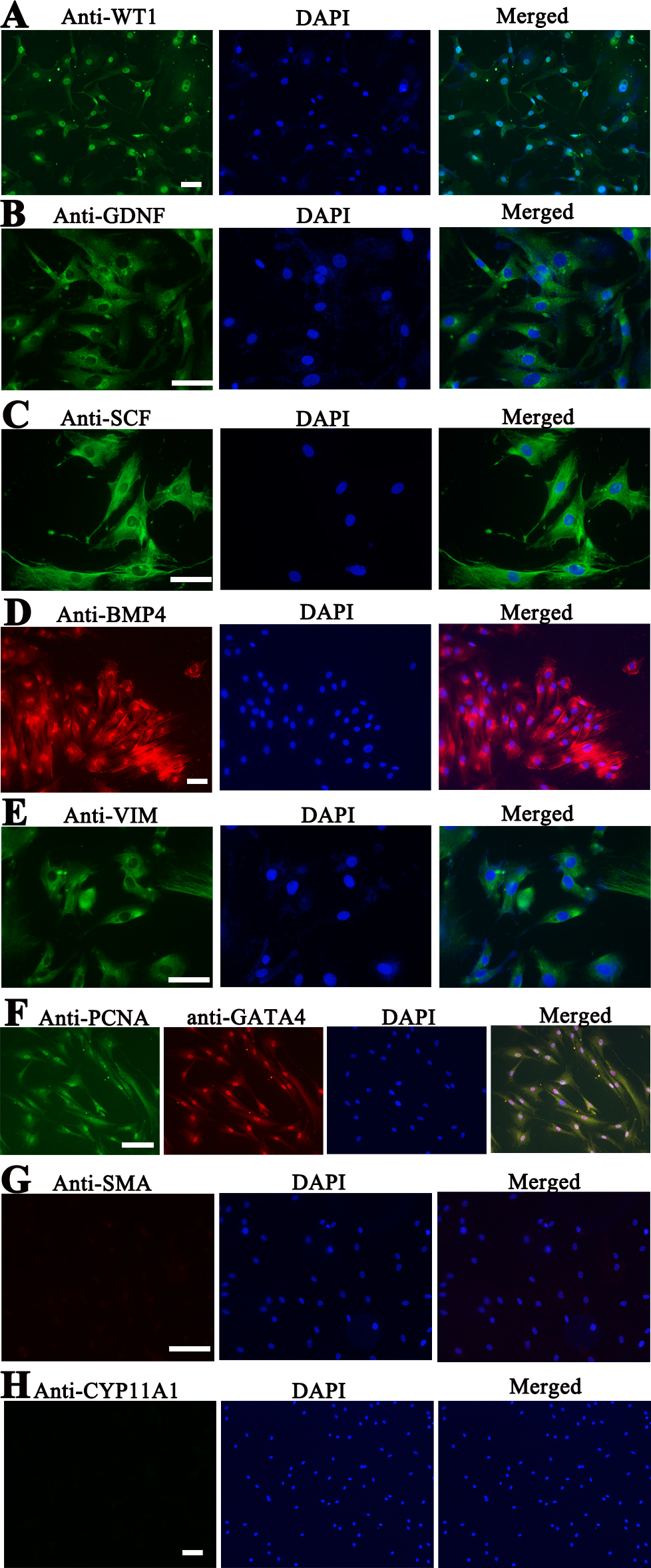

Supplement: Additional file 5: Figure S5. — Expression of a number of proteins after culture for 10 passages. Immunofluorescence revealed the expression of WT1 (A), GDNF (B), SCF (C), BMP4 (D), VIM (E), PCNA and GATA4 (F), SMA(G), and CYP11A1 (H) in human Sertoli cells at passage 10. Scale bars in A, B, C, E, F, G, H = 50 μm; scale bar in D =20 μm. [file 12964_2015_101_MOESM5_ESM.jpeg]
